# A *NOTCH3* homozygous nonsense mutation in familial Sneddon syndrome with pediatric stroke

**DOI:** 10.1007/s00415-020-10081-5

**Published:** 2020-09-26

**Authors:** Elli Katharine Greisenegger, Sara Llufriu, Angel Chamorro, Alvaro Cervera, Adriano Jimenez-Escrig, Klemens Rappersberger, Wolfgang Marik, Stefan Greisenegger, Elisabeth Stögmann, Tamara Kopp, Tim M. Strom, Jörg Henes, Anne Joutel, Alexander Zimprich

**Affiliations:** 1 Department of Dermatology and Venereology, University Hospital of St. Pölten, Karl Landsteiner University of Health Sciences, St. Pölten, Austria; 2 Department of Neurology, Medical University of Vienna, Währinger Gürtel 18-20, 1090 Vienna, Austria; 3 Laboratory of Advanced Imaging in Neuroimmunological Diseases, Center of Neuroimmunology, Hospital Clinic Barcelona, IDIBAPS and Universitat de Barcelona, Barcelona, Spain; 4 Department of Neuroscience, Comprehensive Stroke Center, Hospital Clinic Barcelona, Barcelona, Spain; 5 Institure Investigacions Biomèdicas August Pi I Sunyer (IDIBAPS), Universitat de Barcelona, Barcelona, Spain; 6 Royal Darwin Hospital, Darwin, NT Australia; 7 Department of Neurology, Hospital Ramon Y Cajal, 28034 Madrid, Spain; 8 Department of Dermatology, Rudolfstiftung Hospital, Vienna, Austria; 9 Division of Neuroradiology and Musculoskeletal Radiology, Department of Biomedical Imaging and Image-Guided Therapy, Medical University of Vienna, Vienna, Austria; 10 Juvenis Medical Center, 1010 Vienna, Austria; 11 Institute of Human Genetics, Technical University Munich, Munich, Germany; 12 Department of Internal Medicine II (Hematology, Oncology, Rheumatology and Clinical Immunology), Centre for Interdisciplinary Clinical Rheumatology and Immunology, Eberhard Karls-University Tuebingen, Tübingen, Germany; 13 Institute of Psychiatry and Neurosciences of Paris, INSERM UMR1266, University of Paris, 75014 Paris, France

**Keywords:** NOTCH3, CADASIL, Sneddon syndrome, Homozygous nonsense mutation

## Abstract

Sneddon syndrome is a rare disorder affecting small and medium-sized blood vessels that is characterized by the association of livedo reticularis and stroke. We performed whole-exome sequencing (WES) in 2 affected siblings of a consanguineous family with childhood-onset stroke and identified a homozygous nonsense mutation within the epidermal growth factor repeat (EGFr) 19 of NOTCH3, p.(Arg735Ter). WES of 6 additional cases with adult-onset stroke revealed 2 patients carrying heterozygous loss-of-function variants in putative *NOTCH3* downstream genes, *ANGPTL4,* and *PALLD*. Our findings suggest that impaired *NOTCH3* signaling is one underlying disease mechanism and that bi-allelic loss-of-function mutation in *NOTCH3* is a cause of familial Sneddon syndrome with pediatric stroke.

## Introduction

Sneddon syndrome (SS) is a rare disorder (about 4 patients per million), affecting mainly young and predominately female adults [1,2]. It is characterized by recurrent strokes and livedo reticularis, a violaceous, netlike patterning of the skin [3]. Skin biopsies often display distinct histopathological findings consisting in sequential stage-specific changes in small to medium- sized arteries at the border between dermis and subcutis such as a possibly short-lived endotheliitis, followed by inflammatory obstruction, subendothelial cell proliferation and fibrosis of the occluded artery and shrinkage of the vessel [3–5]. Nevertheless, several cases with SS have also been described showing inconspicuous histopathological results [6]. Apart from cerebrovascular events as the prominent clinical manifestation, the range of associated pathologies varies from migraine and seizures to spontaneous abortion or cardiac and renal involvement [2]. The pathogenesis of SS is still unresolved and a matter of discussion [5]. An association with the occurrence of antiphospholipid antibodies and cofactors is described, although the reported frequencies show a vast range also including cases without any antibodies [7]. One explanatory model proposes that the presence of antiphospholipid antibodies might point towards a thrombotic process causing the disease whereas skin biopsies of antibody negative patients suggest a primary inflammatory process with migration and proliferation of smooth muscle cells leading to the narrowing and occlusion of the vessel [3,8]. Furthermore, it has been suggested that genetic factors also contribute to disease development [9,10]. In 2014, a compound heterozygous mutation in the adenosine deaminase 2 (*ADA2)* gene was identified in a large Portuguese family, who presented with livedo reticularis, stroke during early adulthood, leg ulcerations and intermittent fever [11]. More recently, a homozygous *NOTCH3* nonsense mutation was identified in a patient who exhibited livedo reticularis from birth and childhood-onset cavitating leukoencephalopathy with multiple deep lacunar infarcts, disseminated microbleeds and two saccular aneurysms of middle cerebral arteries [12,13].

*NOTCH3* encodes a transmembrane receptor predominantly expressed in mural cells of small blood vessels that plays a critical role in their integrity [14]. Dominant mutations in *NOTCH3* cause CADASIL, a small vessel disease of the brain that manifests in mid-adulthood with leukoencephalopathy and subcortical ischemic events, progressively leading to disability, cognitive decline and premature death (MIM#125,310) [15]. In our study, we performed whole–exome sequencing (WES) in a consanguineous family with SS and 6 additional unrelated patients to analyze the genetic background of this disease.

## Methods

### Study participants

The diagnosis of SS was made based on the clinical criteria for SS, the occurrence of generalized livedo reticularis and the history of cerebrovascular events.

### Sequence analyses

Whole exome data were generated from individuals III:2 and III:3 of family 1 and from the 6 other SS cases. Exomes were enriched with the SureSelect Human All Exon v6 kit (Agilent Technologies, Santa Clara, USA) and DNA libraries were sequenced on a HiSeq 4000 instrument (2 × 100 cycles, Illumina, San Diego, USA). The average exome coverages ranged from 115 × to 197 × and 100% of the *NOTCH3* region was covered with at least 25 ×. Variants were filtered on the minor allele frequency (MAF < 0.001), which was estimated using the in-house database of the Helmholtzzentrum (> 20.000 exomes) and confirmed by the Genome Aggregation Database (gnomAD). *NOTCH3*, *PALLD* and *ANGPTL4* sequence variants were confirmed by Sanger sequencing using standard protocols.

### Differential gene expression of GSE58368 and GSE55203

To find out differentially expressed genes in Notch3 knock-out (KO) mouse models we analyzed 2 microarray datasets (GSE58368 and GSE55203) derived from the Gene Expression Omnibus (GEO) database (https://www.ncbi.nlm.nih.gov/gds/). Using the GEO2R web tool(https://www.ncbi.nlm.nih.gov/geo/geo2r/) samples from the same cell type were analyzed comparing either heterozygous vs. homozygous (GSE58368) or homozygous vs. wildtype (GSE55203) mice.

## Results

**Fig. 1:**
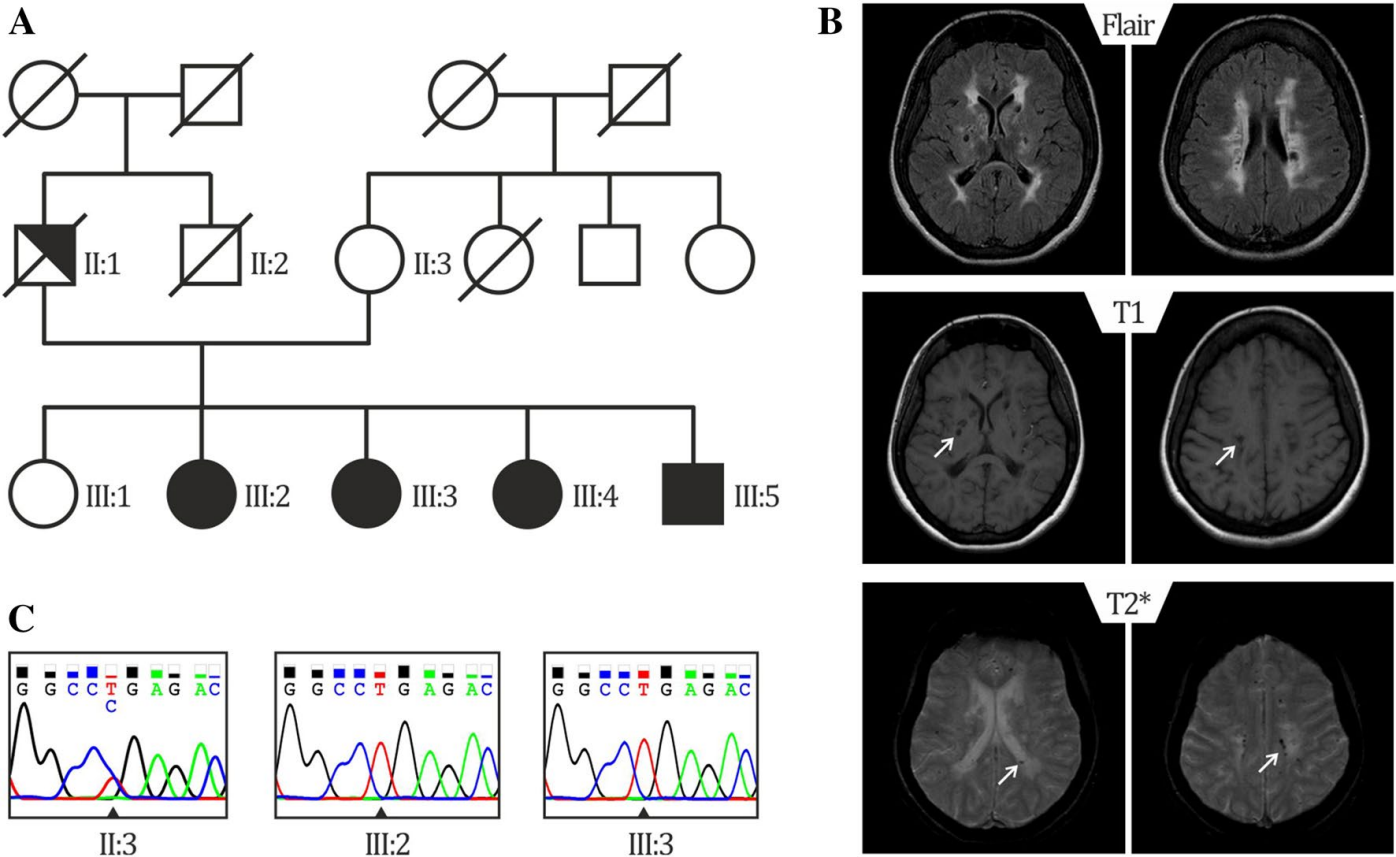
Genealogical tree of the mutated family and representative brain MRI of patient III:3 **a** Pedigree of family 1: Unaffected family members are indicated by open symbols; affected members by closed symbols including livedo reticularis and cerebrovascular manifestations; half closed symbol (II:1) indicates partial phenotype of SS, with only livedo reticularis; diagonal bars through symbols denote deceased individuals. **b** Sanger-Sequence Pherograms showing the *NOTCH3* variant in heterozygous form (II:3) and homozygous form (III:2, III:3). **c** Brain MRI from patient III:3 showing diffuse white matter hyperintensities on fluid-attenuated inversion recovery (FLAIR) images; lacunes on T1 and microbleeds on T2*-weighted images, lesions are depicted by an arrow

**Fig. 2:**
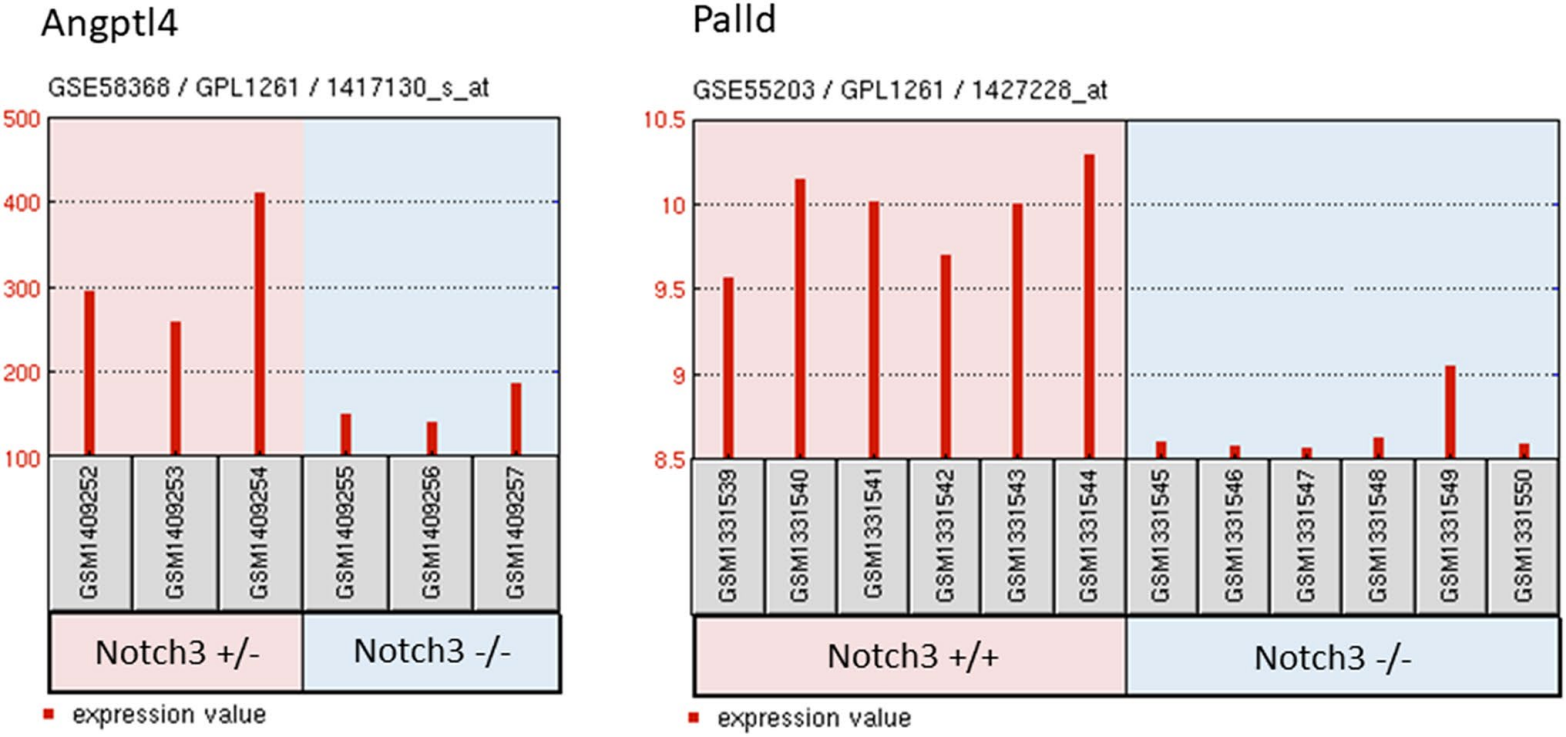
Gene expression profiles GSE58368 and GSE55203 of Palld and Angptl4. Datasets were downloaded from the Gene Expression Omnibus (GEO) database. Data derived from GSE58368, show Angptl4 gene expression in brain derived smooth muscle cells from from Notch3 heterozygote mice (Notch3 ±) compared to cells from Notch3 KO (Notch3 −/−) mice. Data derived from GSE55203 show Palld gene expression in brain microvascular fragments from Notch3 KO (Notch3 −/−) mice compared to those from wild-type (Notch3 + / +) mice

In the present study, we performed WES in 2 patients of a family with SS (Fig. 1a, subject III:2 and III:3). Clinical details of patient III:3 were previously reported [10]. Briefly, of 5 siblings 4 are affected with SS presenting livedo reticularis and a history of early onset stroke in childhood. Brain MRI from subjects III:2 and III:3 showed severe periventricular and subcortical white matter lesions and also multiple microbleeds predominantly in the white matter in sibling III:3 (Fig. 1b and Table 1). Laboratory results of sibling III:3 were negative for antiphospholipid antibodies and thrombophilia in general. No data were available on the antibody profile of the second sibling (III:2). The mother was reported to be healthy and the father, who died from a myocardial infarction at the age of 54, was reported to have had livedo reticularis but no signs of cerebrovascular disease (Fig. 1a). Exome-data analysis revealed in both siblings a large homozygous region of 9 Megabases on chr.19p13., indicating consanguinity. This region harbored only two homozygous variants shared by both siblings. First, a missense variant was detected in the *KANK2* gene p.(Met278Lys), which is not present in any publicly available database. This variant was discarded since mutations in this gene have been associated with a distinct recessive disease characterized by a nephrotic syndrome and palmoplantar keratoderma with woolly hair (OMIM*614610). Second, a homozygous nonsense variant was identified in exon 14 of the *NOTCH3* gene, p.(Arg735Ter) (Fig. 1b), which lies in the Epidermal Growth Factor repeat (EGFr) 19 of NOTCH3. This variant is predicted to result in a premature stop codon and elicit nonsense-mediated mRNA decay [16]. The variant is present heterozygously in 2 out of 61.000 individuals in the gnomAD database. (https://gnomad.broadinstitute.org/). The healthy mother was tested to be heterozygous for the p.(Arg735Ter) variant (Fig. 1c, II:3). No other family member was available for genetic testing either because they lived in another region of the country and were not able to transfer to the clinic for testing or were not interested in participating in the study. Based on the severity of the mutation and the known role of *NOTCH3* in cerebrovascular disease, we believe that this mutation is most probably the cause of the disease in this family. To analyze whether *NOTCH3* loss-of-function (lof) mutations are a more frequent cause for SS, we performed WES in 6 additional unrelated patients (Table 2). We found none of the patients to carry a rare variant (MAF < 0.01) in the *NOTCH3* or in the *ADA2* gene. Moreover, high coverage sequencing allowed us to also exclude copy number variations in both genes. We next hypothesized that genetic variations in genes involved in the *NOTCH3* pathway might be plausible disease candidates. To explore this possibility, we made use of two microarray datasets of *Notch3* KO mouse models from which RNA expression data of brain microvascular fragments (GSE55203) or brain-derived smooth muscle cells (GSE58368) were deposited. Using the GEO2R tool we searched for genes which were significantly downregulated in KO cells compared to wildtype or heterozygous cells (*p* value < 0.01), assuming that these genes are likely downstream in the *NOTCH*3 signaling pathway. We then intersected these 445 genes with the 85 genes carrying lof variants in our 6 patients. We found two patients with lof variants in putative *NOTCH3* downstream genes. Patient 895 carried a heterozygous nonsense variant in the Palladin (*PALLD*) gene, p.(Arg287Ter) and patient 898 carried a heterozygous frameshift variant in the Angiopoietin-like 4 gene (*ANGPTL4*), p.(Gly313AlafsTer49). Both variants are present heterozygously in the gnomAD database (*PALLD*: 10/277212 alleles, *ANGPTL4*: 57/280350 alleles) (Fig. 2, Table 2).

**Table 1:** Main clinical and neuroimaging features of family members

Patient (sex, age in years) *NOTCH3* variant	Livedo reticularis	Age at 1st stroke	Neurological manifestations	Brain MRI	Serology
III:2 (F, 49) p.(Arg735Ter) (homozygous)	Yes His.: n.a	3 months	Small vessel stroke Hemiparesis L; mild cognitive impairment; reduced mobility Syncopes, urinary incontinency, pseudobulbar palsy with dysphagia and unmotivated laughing	Diffuse WMH Multiple lacunar infarctions	n.a
III:3 (F, 41) p.(Arg735Ter) (homozygous)	Yes His.: normal	5 years	Small vessel stroke Hemiparesis R, ataxia, mild to moderate memory problems, progressive impairment of mobility, pseudobulbar palsy with dysarthria and unmotivated laughing	Diffuse WMH Multiple lacunar infarctions Microbleeds Normal MR angiography	Anti-phospholipid antibodies negative
II:1 (M, 54 +) n.a	Yes His.: n.a	None	None	n.a	n.a
II.2 (F, 72) p.(Arg735Ter) (heterozygous)	No	None	None	n.a	n.a
III:4 (F, 47) n.a	Yes His.:n.a	Childhood	Hemiparesis L since childhood, dysarthria, impaired mobility, memory problems	n.a	n.a
III:5 (M, 33) n.a	Yes His.: n.a	2 years	Hemiparesis R	n.a	n.a

*R* right side, *L* left side, *WMH* white matter hyperintensities, *His* histology, *n.a.* not available,  +  deceased

*NOTCH3* complete variant description: *NOTCH3*: g.chr19:15296161G > A (GRCh37/hg19); c.2203C > T (NM_000435.2); p.(Arg735Ter) (rs773299588)

**Table 2:** Main clinical and neuroimaging features of the 6 additional patients with SS

Patient (sex, age in years) Variant	Livedo reticularis	Cerebrovascular events (Age at 1st manifestation in years)	Brain MRI	Other manifestations	Serology
893 (F, 48)	Yes His.: n.a	Multiples ischemic strokes (41)	Small vessel stroke, multiple small ischemic lesions	Arthralgia	ACA, LAC, ANAs
894 (M, 54)	Yes His.: pos^+^	Transient ischemic attacks (47)	Small vessel stroke, multiple small bifrontal WMH	Syncopes, arterial embolus of the left foot, myocardial infarction, coronary microvascular disease, cardiac arrhythmia	ACA, anti-B2GPI, PR3-ANCAs
895 (F, 63) *PALLD* p.(Arg287Ter)	Yes His.: n.a	Multiple ischemic strokes with consecutive epilepsia (49)	Small vessel stroke, multiple ischemic lesions, microbleeds, progressive stenosis of the left V2-segment of the vertebral artery	Retinal vasculitis with vascular occlusions, Temporalis artery occlusion, Arthritis of the wrist joints	LAC, ACA, anti-B2GPI, ANAs
896 (F, 62)	Yes His.: pos^++^	Strokes (L and R hemiparesis) (24)	Small vessel stroke, ischemic lesions	Myocardial infarction, coronary artery disease, sick sinus syndrome, chronic kidney disease, renal insufficiency vasculitis of the toes	ACA, ANAs (intermittent positive)
897 (F, 41)	Yes His: negative	Cerebral vasculitis, migraine attacks (38)	Small vessel stroke, multiple small frontal WMH	Arterial hypertension, syncopes, bradycardia fibromyalgia, fatigue, ulcerative colitis	ACA, anti-B2GPI, HLA B27
898 (F, 42) *ANGPTL4*: p.(Gly313AlafsTer49)	Yes His: negative	Small vessel stroke (39)	Posterior cerebral artery infarction (L); occlusion of the left posterior cerebral artery (P2 segment)	Arterial hypertension, miscarriages	ACA, anti-B2GPI, LAC, Anti-dsDNA and ASMA

*F* female, *M* male, *His* histologym *n.a.* not available, *pos*^+^ early inflammatory stage, *pos*^++^ subendothelial proliferation, *R* right side, *L* left side, *WMH* white matter hyperintensities, *ACA* anticardiolipin antibody, *anti-B2GPI* anti-β-2-glycoprotein 1 antibody, *Anti-dsDNA* anti-double stranded DNA antibody, *ANA* anti-nuclear antibody, *ASMA* anti-smooth muscle actin antibody, *ANCA* anti-neutrophil cytoplasmic antibody, *LAC* lupus anticoagulant

*PALLD and ANGPDL4* complete variant description: *PALLD*: g.chr4: 169433514 C > T (GRCh37/hg19), NM_016081: c. 859C > T p.(Arg287Ter) (rs138149986); *ANGPTL4:* g.chr19: 19:8436302-TG > T (GRCh37/hg19), NM_139314: c.938del: p.(Gly313AlafsTer49) (rs747940485)

## Discussion

Herein, we identified a homozygous nonsense mutation in the *NOTCH3* gene in 2 affected siblings of a consanguineous SS family with pediatric stroke. Remarkably, a *NOTCH3* null mutation was identified in another unrelated patient with similar clinical and MRI features, as well as childhood-onset, originally diagnosed with SS [12,13]. These findings, added to the fact that *NOTCH3* plays a key role in small brain vessels, strongly suggests that loss of NOTCH3 signaling is one underlying disease mechanism for SS.

Although these three patients with SS and a *NOTCH3* null mutation exhibit clinical and neuroimaging features that share similarities with those observed in CADASIL patients, we believe that this genetic form of SS and CADASIL are two distinct entities. First, stroke events started in these 3 SS patients in childhood whereas they occur in CADASIL patients in adulthood, at a mean age of 49 years [15]. Second, livedo reticularis is absent in CADASIL [15]. Third, CADASIL mutations are dominantly inherited and characteristically lead to the loss or gain of a cysteine residue in one of the EGFr of the extracellular domain of NOTCH3 [15]. Fourth, accumulating evidence indicates that CADASIL is not caused by a loss of NOTCH3 function [17–19], but by a neomorphic effect related to the abnormal vascular accumulation of NOTCH3 protein and possibly an increased activity of the mutant receptor [20,21]. Interestingly, patients with NOTCH3 lof mutations like our patient (III:2) and the previous described case [12,13] seem to differ from other SS patients. In addition to the childhood-onset and the more severe disease course, NOTCH3 lof mutation carriers showed negative serum antibody profiles and no extraneurological manifestations. Thus, one might hypothesize that NOTCH3 lof mutations lead to a distinct and probably more severe clinical subtype of SS. In an attempt to find other possible contributing genes in SS patients with adult-onset stroke, we searched for lof variants in genes downstream to NOTCH3. Hereby, we found 2 patients carrying heterozygous lof variants in the *PALLD* and *ANGPTL4* genes. Interestingly, both genes show connections to vascular biology and stroke. *PALLD*, which is predominantly expressed in arterial smooth muscle cells in the brain (https://betsholtzlab.org/VascularSingleCells/database.html), was shown to be involved in the modulation of the actin cytoskeleton and plays a role in vascular remodeling [22,23]. In addition, *PALLD* gene polymorphisms were found to be associated with stroke [24]. *ANGPTL4* was found to be involved in angiogenesis and vessel sprouting in a rat stroke model and was also shown to have a vasculoprotective effect in a mouse stroke model [25,26]. However, both variants *PALLD*-Arg287Ter and *ANGPTL4*- Gly313AlafsTer49 are, although rare, present in the general population with 1 in ~ 14,000 and 1 in ~ 2500 respectively and thus unlikely to cause SS on their own. Therefore the presented candidate genes must only be regarded as suggestions for follow up studies and caution in interpretation is advised. Alternatively, we suggest that they could increase disease susceptibility, possibly in combination with other genetic and/or environmental factors. However, one limitation of our study is that unfortunately other family members of patients 895 and 898 were lost to follow up and segregation of the variants could therefore not be investigated.

In conclusion, we propose *NOTCH3* null mutations as a genetic cause for SS with childhood-onset stroke. We further suggest that impairment of NOTCH3 signaling may also contribute to SS pathogenesis in general.

## Availability of data and material

All raw data and a complete list of all rare sequence variants (MAF < 0.001) generated by WES are available upon request.
